# CO-DESIGNING AN MHEALTH-BASED CAREGIVING MASTERY PROGRAM FOR DELIRIUM PREVENTION IN PERSONS LIVING WITH DEMENTIA

**DOI:** 10.1093/geroni/igae098.3351

**Published:** 2024-12-31

**Authors:** Yong Kyung Choi, Shih-Yin Lin, Kate Santoso, Jennifer Pruskowski, Donna Fick, Abraham Brody

**Affiliations:** University of Pittsburgh, Pittsburgh, Pennsylvania, United States; New York University, New York, New York, United States; NYU Langone Health, New York, New York, United States; University of Pittsburgh, Pittsburgh, Pennsylvania, United States; The Pennsylvania State University, University Park, Pennsylvania, United States; HIGN, New York University Rory Meyers College of Nursing, New York, New York, United States

## Abstract

Caregivers play an essential role in the prevention and early detection of Delirium Superimposed on Dementia (DSD) in community settings. To support caregivers, our study utilized a co-design methodology to adapt an existing evidence-based comprehensive dementia symptom management program originally designed for clinicians (Aliviado Dementia Care) for use by caregivers with a specific focus on DSD (Aliviado DSD Caregiving Mastery). We enrolled 10 caregivers of persons living with dementia (70% female; mean age=57.2; 5 caregivers having previously cared for their care recipient when experiencing delirium) to participate in a 5-week co-design workshop via Zoom. Over the 5 weeks, participants co-designed a series of family education videos, care plans, and communication guides; and refined the existing caregiver education articles and Aliviado mobile app. Session transcripts were analyzed immediately post-session using Instant Data Analysis to generate key themes for improvements. The main themes included caregiver-friendly language across program elements (e.g. changing “non-pharmacological” to “non-medication-based”), clear next steps (e.g. whether to go to the ER, timing to start a new care plan, etc.), and fine-tuning the app’s user interface (e.g. improved navigation between caregiver-administered delirium assessment questions). The co-design approach not only increased person- and family-centeredness of this program but also produced more actionable program components that empower caregivers to deliver DSD care and be better advocates for their loved ones. This collaborative process underscores the importance of engaging caregivers as co-designers, yielding a more accessible and user-friendly set of tools to support caregivers and their care recipients.

